# A pathway care model allowing low-risk patients to gain direct admission to a hospital medical ward − a pilot study on ambulance nurses and Emergency Department physicians

**DOI:** 10.1186/s13049-014-0072-0

**Published:** 2014-12-10

**Authors:** Birgitta Wireklint Sundström, Emelie Petersson, Marcus Sjöholm, Carita Gelang, Christer Axelsson, Thomas Karlsson, Johan Herlitz

**Affiliations:** School of Health Sciences, Research Centre PreHospen, University of Borås, The Prehospital Research Centre of Western Sweden, SE-501 90 Borås, Sweden; Gothenburg EMS System, Sahlgrenska Academy and University Hospital, Gothenburg, Sweden; Department of Public Health and Community Medicine, Sahlgrenska Academy and University Hospital, Gothenburg, Sweden

## Abstract

**Abstract:**

A pathway care model allowing low-risk patients to gain rapid admission to a hospital medical ward − a pilot study on ambulance nurses and Emergency Department physicians.

**Background:**

Patients with non-urgent medical symptoms who nonetheless require inpatient hospital treatment often have to wait for an unacceptably long time at the Emergency Department (ED). The purpose of this study is to evaluate the feasibility and effect on length of delay of a pathway care model for low-risk patients who have undergone prehospital assessment by an ambulance nurse and ED assessment by a physician within 10 minutes of arrival at the ED.

**Methods:**

The pilot study comparing two low-risk groups took place in western Sweden from October 2011 until January 2012. The pathway model for low-risk patients was used prospectively in the rapid admission group (N = 51), who were admitted rapidly after being assessed by the nurse on scene and then assessed by the ED physician on ED admission. A retrospectively assembled control group (N = 51) received traditional care at the ED. All p-values are age-adjusted.

**Results:**

Patients in the rapid admission group were older (mean age 80 years old) than patients in the control group (mean age 73 years old) (p = 0.02). The median delay from arrival at the patient’s side until arrival in a hospital medical ward was 57 minutes for the rapid admission group versus 4 hours 13 minutes for the control group (p < 0.0001). However, the median delay time from the ambulance’s arrival at the patient’s side until the nurse was free for a new assignment was 77 minutes for the rapid admission group versus 49 minutes for the control group (p < 0.0001). The 30-day mortality rate was 20% for the rapid admission group and only 4% for the control group (p = 0.16).

**Conclusion:**

The pathway care model for low-risk patients gaining rapid admission to a hospital medical ward shortened length of delay from the first assessment until arrival at the ward. However, the result was achieved at the cost of an increased workload for the ambulance nurse. Furthermore patients who were rapidly admitted to a hospital ward had a high age level and a high early mortality rate. Patient safety in this new model of fast-track assessment needs to be further evaluated.

## Introduction

In Emergency Medicine different triage models are being used in order to assess and triage systematically and prioritise according to the urgency of care need [1,2]. Triage of patients has previously in most instances been performed in the Emergency Department (ED) by physicians and registered nurses. In the prehospital emergency medical services (EMS) triage of patients has from a historical perspective mostly taken place in connection with major accidents and disasters, where a large number of patients were involved in the prehospital setting [3].

The Rapid Emergency Triage and Treatment System (RETTS) is a five-point model consisting of two parts which in combination result in a priority assessment of the patients. The RETTS triage model is based on vital signs and an Emergency Symptoms and Signs (ESS) code depending on the reason the patient called for help. Objective vital signs including blood pressure, oxygen saturation, breathing frequency, heart rate, body temperature and degree of consciousness result in a triage colour (red, orange, green, yellow and blue) [4].

Of these triage colours, red means ‘life-threatening condition’ indicating immediate emergency hospital care (i.e. vital signs constantly monitored) and the ambulance nurse constantly at the patient’s bedside. Orange means ‘possible life-threatening condition’ also indicating immediate emergency hospital care (i.e. vital signs constantly monitored and the ambulance nurse available but not constantly at the patient’s bedside). Yellow means `not a life-threatening condition’ nevertheless requiring emergency hospital care within a reasonable time (i.e. the patient can wait). Vital signs need to be controlled at intervals. Green means ‘no requirement for care within a reasonable time’ (i.e. patient can wait). There is no need for support or regular controls. Blue means that the patient is not in need of emergency care and can therefore be treated at another care level [4].

## Background

Previous studies on fast-track assessment and various triage models used in the ED by nurses have shown difficulties in triaging patients to the right level of care. The highest accuracy has been reported among those patients who were most disabled but the opposite has also been found [5,6]. The evaluation of the need for hospitalisation showed lowest accuracy among patients with multiple diseases and those with non-specified chest pain [5]. Contradictory findings have been reported concerning whether or not nurses have a higher or lower tendency to admit patients to a hospital ward compared with physicians [5,6]. Clinical cognition and diagnostic error have been reported among physicians [7].

### Pathway care models

Research on fast-track assessment and different pathways is of importance in order to identify ways in which delays to patient care can be safely reduced. A number of different prehospital pathways are relevant for patients suffering from suspected myocardial infarction [8], suspected stroke [9], suspected hip fracture [10] and for geriatric patients [11]. For patients with myocardial infarction or stroke, the time from calling the EMS until delivery of treatment is many times of utmost importance for the outcome [12]. There are two factors in the prehospital setting that are of importance for the outcome. The first is the early identification of the possible primary diagnosis behind the symptoms and the second is the length of the delay between calling the EMS and delivery of the appropriate treatment. Both are equally important [13–15].

The development of pathways for patients without urgent symptoms who nevertheless still require treatment in hospital is explained by observation of these patients who often have to wait for an unacceptably long time in the ED until they are admitted to a hospital ward. By excluding these patients from traditional care at the ED there will hopefully be more room for patients suffering from urgent symptoms [16]. Such findings indicate that fast-track assessment seems to be effective and can minimise delays. Nevertheless, one study has recently shown an evidence gap regarding the real effects of prehospital triage systems, highlighting the lack of knowledge about potential effectiveness [17].

The purpose of this study is to evaluate the feasibility and effect on length of delay of a pathway care model for low-risk patients who have undergone prehospital assessment by an ambulance nurse and ED assessment by a physician within 10 minutes of arrival at the ED.

## Methods

### Design and settings

This study was designed to evaluate a pathway care model in allowing low-risk patients to gain rapid admission to a hospital medical ward. This pilot study attempts to identify whether or not hospitalisation with rapid admission was needed for patients with non-urgent conditions (yellow or less, according to RETTS). If this was the case, the patient had to be transported rapidly to a hospital medical ward. Two groups were compared: the intervention group for rapid admission and the control group. The RETTS triage model was used both in the EMS and at the ED.

This study took place in one of three city hospitals in the municipal district of Gothenburg, Sweden, during four months from October 2011 until January 2012. During those four months, 14,411 patients were transported by the EMS to one or other of the three EDs in Gothenburg. Among them 7,375 were assessed as medical conditions and of these 4,372 were triaged yellow or green according to RETTS.

Patients were only included on weekdays between 08.30 and 19.30. Data sources from the EMS as well as from the hospital were gathered together in the electronic patient care record (ePCR) system. Ethical approval by the Committee for Ethics in Medical Interventions, Gothenburg University, Sweden was applied for, but the study was not judged as requiring ethical approval (no ethical questions).

### Ethical considerations

This study was judged by the ethical review board as a quality improvement project and therefore as not requiring formal ethical approval. The patients’ informed consent for participation was therefore not required formally. Despite this decision all corresponding strict ethical demands have been applied in the entire project implementation. Special attention was paid to ensuring that the encounters with the participating patients as well as their relatives were respectful, especially with consideration to their high age level and corresponding vulnerability. This study was designed to meet the ethical principles for research procedures as put forward by the International Council of Nurses [18], ensuring that the principles of anonymity, integrity and confidentiality are maintained.

Patients’ confidentiality was protected by replacing their civic registration numbers with other numbers thus making identification impossible. We emphasised awareness of and consideration for patient safety in a careful and ambitious documentation, consisting of much data about the patients’ care and treatment during the entire pathway, prehospital as well in hospital.

### The intervention team in the EMS

The intervention team consisted of 21 ambulance nurses in the prehospital EMS who were specially delegated to carry out prehospital assessment and take decisions about rapid admission for low-risk patients. They were professionally qualified as registered nurses and the majority had specialist education in prehospital emergency care (ambulance services) or in anaesthetic care. One had specialist education in intensive care, one had two specialist educations and one had no specialist education. In total they each had at least 4 years’ experience in the prehospital EMS (Table 1).

**Table 1:** **Socio-demographic and professional characteristics of the intervention team (N = 21)**

***Sex***	
Male	11
Female	10
*Number of years in ambulance service*	
≥4	7
5-9	7
10-14	5
15-32	2
*Education*	
Registered Nurse (RN)	1
RN with specialist education in prehospital emergency care (ambulance service)	12
RN with specialist education in anaesthetic care	6
RN with specialist education in intensive care	1
RN with specialist education in prehospital emergency care and anaesthetic care	1

### The pathway care model for low-risk patients

**Figure 1:**
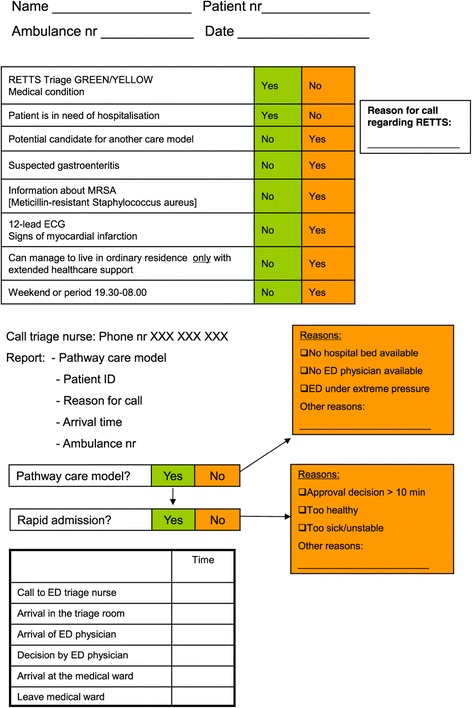
**Flow chart of the pathway care model for low-risk patients.**

The pathway care model (Figure 1) for low-risk patients was used prospectively and based on the ambulance nurses’ assessments on scene. A checklist was included for assessment of the medical condition and the need for hospitalisation, i.e. need of care in a hospital medical ward. Fast-track assessment was thus based on dialogue with the patients/the relatives, with the addition of the patients’ case histories (anamneses) and information on how their health conditions were affected in general, with the support of the checklist.

The checklist is also a support for the documentation where the ambulance nurse records the times for the following events: the call to the ED triage nurse, arrival in the triage room at the ED, arrival of the ED physician, decision by the ED physician, arrival in the hospital medical ward and finally the time when the ambulance nurse leaves the hospital ward and is ready for a new assignment.

The pathway care model for low-risk patients is divided into five steps:

- Step 1: Identification. Patients with a medical condition triaged yellow or green according to RETTS, where the ambulance nurse assesses the patient as requiring hospitalisation. With the support of RETTS, each patient is given an ESS code, based on symptoms and vital signs.
- Step 2: Decision by triage nurse. The ambulance nurse calls the ED triage nurse and reports. The ED triage nurse then calls the ED physician, i.e. the medical specialist.
- Step 3: Arrival in the triage room*.* Approval is given by the ED physician immediately upon arrival at the ED. The ambulance nurse reports and ED assessment is carried out by the medical specialist.
- Step 4: Decision by ed physician*.* A final decision is taken by the medical specialist to admit the patient rapidly to a hospital medical ward.
- Step 5: Arrival at the medical ward. The ambulance nurse transports the patient to the medical ward, where the ambulance nurse reports to the nurse in charge.

Logistical problems and further perspectives on the pathway care model:

- The reasons why the ED triage nurse might decide not to accept the patient for direct admission might be: that there is no hospital bed available, or that there is no medical specialist available or that the ED is under extreme pressure with too much to do at the time.
- The time for the decision by the medical specialist must not exceed 10 minutes. If it does the patient will be excluded from the pathway.
- The medical specialist can decide not to admit the patient to a hospital ward directly if the patient is assessed as being too healthy, too sick or unstable.

### Inclusion and exclusion criteria

The inclusion criteria were: Patients who were seen and 1) assessed by an ambulance nurse (member of the intervention team), 2) as having medical condition yellow or green (according to RETTS, 3) as being in need of hospitalisation and 4) as having received an approval decision by ED physician within 10 minutes.

The exclusion criteria were: 1) Age less than 16 years old, 2) patient suitable for other prehospital clinical pathways, 3) suspicion of gastroenteritis and 4) suspicion of myocardial infarction.

Patients with an estimated delay of more than 10 minutes in the ED were excluded from the pathway care model but are still included in the rapid admission group in the analyses (intention to treat).

### Study population of low-risk patients

The rapid admission group prospectively included patients whose medical condition identified them as low-risk patients, i.e. they were triaged yellow or green according to RETTS, thus having non-urgent conditions. Then an ambulance nurse in the intervention team assessed the patient as being in need of hospitalisation and the patient could therefore be admitted rapidly to a hospital medical ward.

The control group was taken care of according to traditional care at the ED and was assembled retrospectively by use of the ePCR. This meant that patients were triaged yellow or green by an ambulance nurse who was not part of the intervention team. These patients were admitted to the ED during the same time period as patients in the rapid admission group. On admission to the ED they were seen by the ED triage nurse and an ED physician and were thereafter admitted to a hospital ward according to the usual care routines. Thus, inclusion and exclusion criteria were the same for the control group with the exception that the decision about hospitalisation was made by the ED physician, not by the ambulance nurse on scene.

### Sampling strategy

During the time of inclusion (four months) there were 21 ambulance nurses who were authorised to triage patients who were expected to be taken to one of the three city hospitals in Gothenburg for rapid admission to a medical ward. Thus, when the nurses were on duty during weekdays and in daytime, they considered every single low-risk patient for eventual inclusion in the study. Any deviation from this strategy was not recorded.

The sample strategies for including patients in the control group were retrospective via the ePCR system. Consecutive patients, who fulfilled the inclusion criteria and did not have any exclusion criteria, were admitted to the hospital (same as for rapid admission group) and hospitalised in a medical ward. Any deviation from this strategy was not recorded.

### Statistical methods

The primary endpoint of the study was the delay from the EMS’ arrival at the patient’s side until the patient’s arrival in a hospital medical ward. Except for age itself, where the Mann–Whitney U test was used, all comparisons between groups were made using logistic regression for dichotomous variables and a stratum-adjusted Kruskal-Wallis test for continuous/ordered variables to calculate age-adjusted p-values. Percentages, means and medians are presented as crude results (i.e. not age-adjusted). All tests were two-sided and p-values below 0.05 were considered statistically significant. All p-values are age-adjusted and all analyses were performed using SAS 9.3 software.

The hypothesis was that the time from the arrival of the EMS until the patient passes the door to the ward should be reduced from six hours (based on previous calculations) to 1 hour. The power calculation indicated that in order to show this difference if the standard deviation was four hours (at a 5% level) we needed 19 patients in each group.

## Results

In total 102 low-risk patients were included during the study period: 51 patients in the rapid admission group and 51 patients in the control group. Among the 51 patients in the rapid admission group, 45 were directly admitted to a ward (88%). Of the remaining cases, 5 were not evaluated by the ED physician due to logistical problems and 1 was evaluated by the ED physician and assessed as unsuitable for hospital admission.

### Baseline findings

The patients in the rapid admission group were older (Table 2). Among these patients, 12-lead ECG was more frequently recorded than in the control group. In terms of initial priority and length of initial delay, no significant difference was found between the two groups. The length of delay between call received at the Dispatch Centre (Call in) and the EMS’ arrival at the patient was around 30 minutes in both groups. Similar results were also found regarding the length of delay between call from the Dispatch Centre (Call out) to the EMS and the EMS’ arrival at the patient, with 19 minutes in the rapid admission group and 16 minutes in the control group.

**Table 2:** **Baseline characteristics for rapid admission (RA) group and control group (CG)**

	**RA (n = 51)**	**CG (n = 51)**	p*
Age; mean ± SD	80 ± 11	73 ± 16	0.02
Women; %	53	61	0.21
Priority; %			
The Dispatch Centre			0.52#
1	22	20	
2	61	63	
3	18	18	
EMS			0.57
2	39	45	
3	61	55	
Triage colour; %			0.13
Green (RETTS)	31	20	
Yellow (RETTS)	69	80	
12-lead ECG recorded; % (0/5)##			
Delay (minutes); median 25th, 75th percentile)			
Call in – Call out (2/0) ###	11 (4,26)	7 (3,20)	0.57
Call out – arrival at patient	19 (11,25)	16 (11,24)	0.60
Call in – arrival at patient (2/0)	28 (16,43)	0.43	

*age-adjusted (except for age itself).

#priority as an ordered variable used in p-value calculations.

##number of missing in the two groups, respectively.

###call in = call received at the Dispatch Centre from patients/relatives; call out = call from the Dispatch Centre to the EMS.

### Reason for contact

The reasons for contact with the EMS were similar in the two groups. The four most frequent reasons for contact were ‘non-specific disease, dyspnoea, chest pain and vertigo’ (Table 3).

**Table 3:** **Comparison of Emergency Symptoms and Signs (ESS) code in rapid admission (RA) group and control group (CG)**

		**RA (n = 51)**	**CG (n = 51)**
ESS-code; n (1/0)##			
1	Arrhythmia	1	5
2	Hypertension	1	2
4	Dyspnoea	7	8
5	Chest pain	5	7
9	Seizures	0	1
11	Vertigo	6	6
12	Stroke	4	2
15	Pain in extremity	5	3
16	Urinary tract problems	1	0
18	Fatigue/Falling	2	1
19	Headache	0	1
20	Syncope	2	3
47	Infection	3	2
49	Diabetes	1	0
53	Non-specific disease	12	10

## number of missing in the two groups, respectively.

### Status on admission

There was no significant difference between the two groups in terms of various aspects of status on admission by the EMS. Furthermore complications requiring treatment during transport were similar in the two groups (Table 4).

**Table 4:** **Comparison of status on admission in rapid admission (RA) group and control group (CG)**

	**RA (n = 51)**	**CG (n = 51)**	p*
Oxygen saturation (%); mean ± SD (1/1)##	96 ± 3	95 ± 4	0.90
Systolic blood pressure (mmHg); mean ± SD(1/2)	147 ± 27	153 ± 32	0.13
Heart rate (beats/min); mean ± SD (1/1)	84 ± 16	18 ± 3	0.64
Rate of breathing; mean ± SD (4/1)	18 ± 4	18 ± 3	0.96
Pale and/or cold sweat; % (1/0)	14	10	1.00
Nausea and/or vomiting; % (1/0)	6	4	1.00
Presence of pain; % (1/0)	20	14	0.43
Complications during transport requiring treatment; % (4/1)	11	12	1.00

*age-adjusted.

##number of missing in the two groups, respectively.

### Length of delay

The median delay from the arrival of the EMS at the patient’s side until arrival in hospital was shorter in the control group (29 minutes versus 47 minutes in the rapid admission group, p < 0.0001) (Table 5). The overall median delay from the arrival of the EMS until arrival in the hospital ward was 57 minutes in the rapid admission group and 4 hours and 13 minutes in the control group (p < 0.0001); a difference of more than 3 hours.

**Table 5:** **Comparison of length delay in rapid admission (RA) group and control group (CG)**

	**RA (n = 51)**	**CG (n = 51)**	p*
Time from arrival at patient’s side (minutes); mean (25:e,75:e percentile)			
Telephone contact with triage nurse in ED (3/--)##	29 (20,40)		
Arrival in triage room (1/0)	47 (35,55)	29 (24,37)	<0.0001
Arrival of medical specialist (7/--)	50 (37,58)		
Assessment by medical specialist (7/--)	52 (41,60)		
Arrival at hospital ward (11/0)	57 (47,65)	253 (165,383)	<0.0001
Ambulance nurse leaves hospital ward (11/--)	64 (56,72)		
Ambulance nurse available for a new assignment (2/0)	77 (69,86)	49 (42,60)	<0.0001

*age-adjusted.

##number of missing in the two groups, respectively.

However, the median delay from the EMS’ arrival at the patient’s side until the EMS staff had completed their assignment and were available for a new call was significantly prolonged in the rapid admission group (77 minutes versus 49 minutes in the control group, p < 0.0001).

### Events in hospital

The incidence of various complications during the first 24 hours and during hospitalisation did not differ significantly between the two groups (Table 6). However, hospital mortality as well as mortality during the first 30 days was high in the rapid admission group. However, among the 10 patients in this group who died during the first 30 days, 6 were over 90 years of age.

**Table 6:** **Comparison of clinical courses in rapid admission (RA) group and control group (CG)**

	**RA (n = 51)**	**CG (n = 51)**	p*
Complications requiring treatment during first 24 hours; % (10/0)##	32	33	0.20
Complications associated with main diagnosis during hospital stay which required treatment; % (8/1)	33	44	0.12
Hospital stay (days); median (25:e,75:e percentile) (5/0)	7 (5,14)	5 (3,10)	0.27
Mortality;			
In hospital (2/0)	7	0	0.06
In hospital (2/0)	10	4	0.16

*age-adjusted.

##number of missing in the two groups, respectively.

### Final diagnosis

A similar distribution of different diagnoses explained the hospitalisation in the two groups (Table 7). The two most frequent diagnoses were infections and neurological diseases (non-specific disease).

**Table 7:** **Comparison of final diagnoses in rapid admission (RA) group and control group (CG)**

	**RA**	**CG**
Final diagnoses (n) (5/0)##		
Infection	7	8
Malignancy	2	3
Anaemia	1	0
Psychiatric disease	3	5
Neurologic disease	8	8
Peripheral artery disease	6	3
Ischemic heart disease	3	4
Arrhythmia	1	6
Heart failure	3	4
Cerebrovascular disease	2	2
Chronic obstructive disease	2	1
Non-specific disease	8	7

##number of missing in the two groups, respectively.

### Protocol violations

Six patients were assessed by the ambulance nurses as suitable for direct admission but were rejected by the ED triage nurse. Five of them were later admitted to a hospital ward and are therefore included in the rapid admission group. Thus, not all patients in the rapid admission group received an approval decision from the ED physician within 10 minutes as recommended in the protocol.

Three patients assessed by the ambulance nurse as suitable for direct admission were rejected by the ED physician. One of them was later admitted to a hospital medical ward and is therefore included in the rapid admission group.

Eight patients were assessed by the ambulance nurse as unsuitable for direct admission but were later admitted to hospital according to standard care procedures. They were therefore included in the control group.

## Discussion

The main finding in this study is that if a specialist-educated and experienced ambulance nurse had already made a decision that a patient (yellow or less according to RETTS) was in need of hospitalisation, prior to arrival in hospital, then the hospitalisation process could be shortened dramatically. However, there are a number of findings and methodological considerations in this study that need to be addressed.

With the exception of age the two groups were similar at baseline with regard to baseline factors measured. These included the priority given by the Dispatch Centre, the risk assessment made by the ED triage nurse, various delays until arrival at the patient’s side, the ESS code allotted by the ambulance nurse, various aspects of status on admission and the incidence of various complications prior to hospital admission. These findings suggest that it is still meaningful to compare the two groups in terms of outcome data.

The primary endpoint of the study was the delay from the EMS’ arrival at the patient until the patient’s arrival in a hospital medical ward. This delay was reduced by 3 hours in the rapid admission group. Such a reduction is of course dependent on local factors, i.e. the logistics in the local hospital where the study took place [17,19,20]. Therefore our results cannot be extrapolated to other hospitals either inside or outside Sweden.

Our findings, nevertheless, highlight the fact that a fast-track assessment and pathway care model in the prehospital EMS can shorten the waiting time for defined subsets of patients at the ED [21] and no negative impact such as longer waiting time for patients needing immediate treatment has so far been reported [22,23]. On the other hand, increased demands are made on the hospital ward clinicians and patient flow from the ED. Therefore, prehospital triage systems should also evaluate impact on the ED [24] and the hospital wards [2,25].

The median delay from arrival at the patient’s side until arrival in hospital was 18 minutes longer in the rapid admission group. This highlights the fact that the assessment and triage procedure carried out by the ambulance nurse introduced an element of delay prior to arrival in hospital, as described for acute myocardial infarction in women [26] and for trauma [27]. Longer decision time appears to be the consequence of fast-track assessment and prehospital pathways. These results emphasise awareness of the effectivity that is required and that has been discussed [17] although they may also be seen to demonstrate the difficulties in showing all outcomes, measurable as well as not measurable. More research is needed, not least qualitative studies that evaluate the patients’ satisfaction and trust in pathway care models.

The intervention introduced an increased burden of responsibility and workload for the experienced ambulance nurse in the intervention team. There was a further delay of about half an hour before ambulance nurses became available for a new assignment. It is reasonable to assume that with more knowledge and with more experience of the pathway care model, nurses will communicate with the ED triage nurse more effectively and that they will reach a common decision more rapidly as described for triage nurses at the ED [28]. However, according to our findings, it seems that the feasibility of the pathway care model for low-risk patients includes more than assessments based on vital signs and supported by the RETTS triage model, it also contains a meaningful dialogue with the patient [28,29]. The checklist provides support for the ambulance nurses as they question patients about how their general health condition has been affected, focusing on daily living and assessing the possibilities for the patients to return home again after treatment.

There was no significant difference between the two groups regarding short-term mortality. However, there was a slight trend towards a higher mortality in the intervention group during the first 30 days. From a theoretical point of view one might argue that rapid admission might delay a thorough investigation by a physician, once the patient has arrived in the hospital medical ward. We did not find that any of the deaths during the first 30 days could be considered as being associated with delayed or inappropriate treatment in the early phase after hospital admission. However, final diagnosis for these patients included a broad spectrum of diseases some of which were indeed life-threatening. The extraordinary difficulties in predicting which patients need rapid admission is a reflection of the fact that practice in the prehospital EMS is characterised by a wide variety of patient groups and pathologies, as well as extremely varied severity of complaints and conditions [19,30]. Further studies are needed on patient outcomes, including questions on how patient safety is secured.

### Methodological considerations

In this study we used an artificial control group. This means that the control group was not based on the ‘randomised clinical trial’ principle. In accordance with Goodacre (19), we state that important research questions generated by Emergency Medicine are often complex and clinical trials are not always possible, or even the right method to use. Instead the control group was based on consecutive patients who were hospitalised and assessed as low-risk (yellow or green according to RETTS) by the ambulance nurse prior to hospital admission. However, the composition of the control group did not take into account whether or not the ambulance nurse had already assessed that the patient should be hospitalised before hospital admission. It is most probable that the control group consisted of a mixture of patients, some of whom had been assessed as candidates for hospitalisation before their admission to hospital, and some of whom had not.

On the other hand we must admit that even the patients in the rapid admission group consisted of a mixture since not all of them had been assessed by the ED physician as being candidates for hospitalisation. However, the latter group was quite small. And indeed all the patients in the rapid admission group had been assessed by the ambulance nurse as being candidates for hospitalisation.

The rapid admission group was 7 years older (on average) again highlighting that the two groups were not strictly comparable. This was the reason why all p-values were age-adjusted.

### Limitations of the study

The selection of patients in the intervention and the control groups was not identical. The former only included patients assessed by the ambulance nurse as being suitable for rapid admission to a hospital medical ward, but this was not the case for the control group.

The most critical time, i.e. the delay before being assessed and simultaneously treated by a physician at a hospital medical ward, was not assessed. Furthermore, the accuracy in assessments made by the experienced ambulance nurses in the intervention team and the accuracy in assessments made by less experienced ambulance nurses is expected to differ. This indicates a safety limitation for this pilot study, which should be considered in a larger study. Another safety aspect is the high mortality rate in the rapid admission group. Although it did not differ significantly it raises concern and this needs to be carefully evaluated in further studies. Due to the small number of patients in this pilot study the power to detect anything but quite large differences was low.

## Conclusion

The pathway care model for low-risk patients gaining rapid admission to a hospital medical ward shortened length of delay from the first assessment until arrival at the ward. However, the result was achieved at the cost of an increased workload for the ambulance nurse. Furthermore patients who were rapidly admitted to a hospital ward had a high age level and a high early mortality rate. Patient safety in this new model of fast-track assessment needs to be further evaluated. Thus, an out-of-the-ordinary change in healthcare development like this, involving a fast-track assessment for low-risk patients’ needs further scientific evaluation before it can be recommended.
